# Assessment of Activity Profiles in Older Adults and Lower Limb Bone Parameters: Observations from the Hertfordshire Cohort Study

**DOI:** 10.1007/s00223-022-00953-5

**Published:** 2022-02-25

**Authors:** C. M. Parsons, E. M. Dennison, N. Fuggle, M. Ó. Breasail, K. Deere, K. Hannam, J. H. Tobias, C. Cooper, K. A. Ward

**Affiliations:** 1 MRC Lifecourse Epidemiology Centre, Human Health and Development, University of Southampton, Southampton, SO16 6YD UK; 2 MRC Nutrition and Bone Health Research Group, Cambridge, UK; 3 Musculoskeletal Research Unit, University of Bristol, Bristol, UK; 4 National Institute for Health Research Biomedical Research Centre, University of Southampton and University Hospital Southampton NHS Foundation Trust, Southampton, UK; 5 National Institute for Health Research Musculoskeletal Biomedical Research Unit, University of Oxford, Oxford, UK

**Keywords:** Physical activity, Ageing, Epidemiology, pQCT, Accelerometry, Muscle

## Abstract

As muscle strength and function decline with age the optimal high-impact physical activity (PA) required for bone remodelling is rarely achievable in older adults. This study aimed to explore the activity profiles of community-dwelling older men and women and to assess the relationship between individual PA profiles and lower limb bone parameters. Participants from the Hertfordshire Cohort Study wore triaxial accelerometers for 7 days and counts of low (0.5–1.0 g), medium (1.0–1.5 g), and high (> 1.5 g) vertical-impact activity were calculated. Two years later, participants underwent a pQCT scan of the tibia (4% and 38% sites) to obtain measures of bone mineral density and bone geometry. Linear regression was used to quantify associations between bone and PA loading profiles adjusting for age, sex, loading category, and BMI. Results are presented as β [95% confidence interval]. Bone and PA data were available for 82 participants. The mean (SD) age at follow-up was 81.4(2.7) years, 41.5% (*n* = 34) were women. The median low-impact PA count was 5281 (Inter-quartile range (IQR) 2516–12,977), compared with a median of only 189 (IQR 54–593) in medium, and 39 (IQR 9–105) in high-impact counts. Positive associations between high-impact PA and cortical area (mm^2^), polar SSI (mm^3^), and total area (mm^2^) at the 38% slice (6.21 [0.88, 11.54]; 61.94 [25.73, 98.14]; 10.09 [3.18, 16.99], respectively). No significant associations were found at distal tibia. These data suggest that maintaining high (> 1.5 g)-impact activity is difficult for older adults to achieve; however, even small amounts of high-impact PA are positively associated with selected cortical bone parameters 2 years later.

## Introduction

Numerous studies have shown the positive benefits of physical activity (PA) on musculoskeletal health, including prevention of falls, a reduction in fracture risk, maintenance of physical capability, and increased bone mineral density (BMD). The ‘mechanostat’ hypothesis theory states that forces generated through the mechanical loading of muscles influences bone structure by driving bone adaptations through changes in mass and architecture of the bone [1]. In vivo, it has been estimated that at least 1500 microstrains, typically generated by weight bearing PA, are required to drive positive changes in bone [2]. Ideally the activity would impose dynamic, high-magnitude loads applied at a rapid rate and in differing directions, such as football/soccer, tennis, and basketball [3], rather than non-weight bearing activities, such as cycling and swimming.

Until accelerometers were developed, questionnaires were used to estimate the degree of loading to the skeleton through quantification of PA [4, 5]. This proxy measure of mechanical loading was often obtained using self-reported PA in older adults [6]. More recently, an accelerometer-based digital method has been developed [7] and validated [8]. In this method, accelerometers are attached to the trunk and characterise mechanical strain according to vertical impacts. Such data provide a much better characterisation of the loading patterns to which the skeleton is subjected through activity.

The positive benefits of differing physical activities to bone have been widely investigated. Elhakeem and colleagues findings suggested that walking and weight bearing exercise, such as tennis and dancing, may be important for better skeletal health in older age [5]. The benefit of resistance training on skeletal health has also been demonstrated. A meta-analysis by Martyn-St James and Carroll demonstrated that resistance training in women appeared effective in reducing postmenopausal bone loss at the hip and spine [9].

In the United Kingdom (UK) the National Health Service (NHS) recommends that muscle-strengthening, such as cross-training machines or exercising with resistance bands, activities are completed at least twice a week from the age of 35 onwards to prevent natural bone loss [10]. However as we age there is an age-related loss of muscle force-generating capacity and power, including a loss of muscle mass and increase in intramuscular fat. This means that participating in PA that would generate strains required to prevent bone loss or increase bone formation becomes increasingly difficult for older adults [11, 12]. This was demonstrated in older adults where vertical activity counts above 3 g were rarely recorded, showing the predominant form of activity in later life is low impact [13]. Therefore as higher-impact activities become less feasible with age the advice to participate in high-impact activities may be having a demotivating effect on older adults to complete any PA [12]. Despite this there have been many studies aiming to develop high-impact PA programmes which are effective and well tolerated in older adults. Few studies have explored habitual levels of PA in older adults, and the effect these habitual levels have on bone strength. Therefore, our study aimed to determine the activity profiles of community-dwelling older men and women in the UK using accelerometers and to assess the relationship between individual PA profiles and lower limb bone parameters.

## Methods

### Study Participants

The Hertfordshire Cohort Study (HCS) is a large, prospective population-based cohort study and has previously been described in detail [14, 15]. In brief the HCS was originally set up to study origins of adult disease across the lifecourse. Study participants are community-dwelling men and women in the United Kingdom (UK) who were born in the UK county of Hertfordshire between 1931 and 1939 and for those still living within the county baseline recruitment took place between 1998 and 2004 (1579 men and 1418 women). Since their first contact, HCS study participants have continued to take part in various follow-ups detailing their sociodemographic, lifestyle, medical, and biological attributes. In 2014–2015, a subset of HCS baseline participants, residing in East Hertfordshire and who were previously included in the UK arm of the European Project on Osteoarthritis (EPOSA) [16] were approached and invited to participate in the Vertical Impacts in Bone (VIBE) study, a collaborative study with researchers in Bristol, London and Manchester [17]. Of this subset, 274 participants were approached and 143 study participants were provided with wearable activity accelerometers (with useable data obtained from 118 study participants). A further 2 years later, participants were invited to attend a follow-up assessment in which a peripheral quantitative computed tomography (pQCT) scan was completed.

### Physical Activity Accelerometry

As part of the VIBE study, HCS participants received an activated GCDC × 16-1c triaxial accelerometer (Gulf Coast Data Concepts, Waveland, Mississippi, USA) in the mail. Participants were asked to wear the monitor on a custom-designed size-specific elasticated belt over their right hip over a 7-day continuous period for a minimum of 10 h a day, only removing the monitor for sleeping, washing, and swimming. Accelerometers were configured to a sampling frequency of 50 Hz, and raw accelerometry data were imported to Stata 13 (STATA Corp, College Station, Texas, USA) for standardised processing using custom-designed code developed by Deere et al. [7]. A protocol to clean the activity data and then to condense into distinct impact band groups has been previously published by Deere et al. [18]. In brief data cleaning *Y*-axis acceleration peaks were identified based on accelerations which were higher than the preceding and subsequent reading and recorded within 14 pre-specified g bands. These g bands were then condensed further to group all vertical-impact peaks into three distinct bands per participant [7]. These bands reflect low-impact PA peaks between 0.5 and 1.0 g, medium-impact peaks between 1.0 and 1.5 g, and high-impact peaks above 1.5 g [19].The higher-impact cut-point of > 1.5 g was selected as very few impacts were observed within higher g bands [7, 17, 18]. Low-impact activity peaks tend to be associated with activities, such as walking, whereas an aerobics class would produce high-impact PA peaks (above 1.5 g) [13]. Thus, following processing, each individual 7-day activity data were collapsed into 3 variables, a count for the number of times they achieved a vertical-impact load within a low-impact PA peak band, the number of times they achieved a vertical impact within a medium-impact PA peak band, and the number of times they achieved a vertical impact within a high-impact PA peak band.

### Assessment of Bone by pQCT

Data collection was single centre, MRC Elise Widdowson Laboratory, Cambridge, UK. Three operators acquired the scans; all were trained (by KW) and followed standard operating procedures for acquisition. PQCT scans of the non-dominant tibia were performed using a Stratec XCT2000 scanner (Stratec Medizintechnik, Pforzheim, Germany). The tibial length was measured from the distal edge of the medial malleolus to the medial tibial plateau (mm) and slices were taken for the tibia scan at 4% distal tibia and 38% tibial midshaft. At the distal 4% site, CALCBD C1P1, a threshold 180 mg/cm^3^ was used to separate bone from soft tissue, and trabecular from cortical to subcortical bone. At 38% site, CORTMODE 1 with a default threshold of 710 mg/cm^3^ was used for cortical outcomes; 280 mg/cm^3^ for polar strength strain index (SSI).

The CV of the centre for pQCT was calculated from repeat measures of 30 adults and is as follows: trabecular BMD = 0.9%; cortical BMD = 0.7%; and cortical CSA = 1.5%. The quality assurance for pQCT was performed on all working days and the trabecular attenuation and Tot CSA determined for the standard and cone phantoms were always within the accepted tolerance for both pQCT systems. All pQCT scans were scrutinised for movement artefacts and other potential problems to ensure that the scans were of sufficient quality to be included in the study [20].

### Statistical Analysis

Characteristics of study participants were described using means and standard deviations (SD). Each low-, medium-, and high-impact vertical-impact count variables were positively skewed and were log-transformed; medium- and high-impact PA counts included zeros and therefore a count of 1 was added to each study participant’s medium- and high-impact activity count to enable a log transformation to be performed.

The three distinct PA vertical-impact variables were highly correlated and so to account for multi-collinearity residuals were derived for inclusion in regression models by adjusting: low-impact PA counts for medium- and high-impact counts; medium-impact PA counts for low- and high-impact counts; and adjusted for medium and low-impact PA counts.

Linear regression was used to examine the associations between individual pQCT (outcome) and each low-, medium-, and high-impact vertical-impact PA count (exposure) variables, as assessed by accelerometry. Men and women were pooled in these analyses as sex-interaction terms were not statistically significant. Given the close associations between BMI, PA, age, sex, and bone, we have run both unadjusted linear regression models and fully adjusted linear regression models, accounting for age at pQCT scan, sex, BMI and PA. Results are presented as β [95% confidence interval], with positive associations indicating that greater physical impact counts are associated with higher value of the bone parameters.

All analyses were undertaken using Stata, release 14.0 (STATA Corp, College Station, TX, USA) [21].

## Results

Vertical impact PA and pQCT data were available for 82 study participants and a description of these participants is shown in Table 1. At the time of pQCT scan participants were just over 81 years and 58.5% (*n* = 48) of the study population were men and the majority of study participants report having never smoked or being an ex-smoker (98.8% never/ex). Twenty-six percent of women in the study sample reported having osteoporosis and just under 13% of men.

**Table 1:** Study population descriptive statistics at pQCT scan

	All (*n* = 82)		Men (*n* = 48)		Women (*n* = 34)	
	Mean	SD	Mean	SD	Mean	SD
Age (years)	81.4	2.7	81.4	2.7	81.3	2.6
Height (cm)	167.7	9.2	173.3	6.9	159.8	5.7
Weight (kg)	76.3	14.3	82.5	14.1	67.7	9.5
BMI (kg/m^2^)	27.1	4.1	27.5	4.3	26.6	3.8

	*n*	%	*n*	%	*n*	%
Social class						
Non-manual	40	51.3	20	45.5	20	58.8
Manual	38	48.7	24	54.6	14	41.2
Smoking status						
Never	40	49.4	21	44.7	19	55.9
Ex	40	49.4	25	53.2	15	44.1
Current	1	1.2	1	2.1	0	0
Osteoporosis	15	18.5	6	12.8	9	26.5

	Median	IQR	Median	IQR	Median	IQR
Number of comorbidities^a^	2	1–4	2	1–3	3	1–4

^a^Comorbidities considered were high blood pressure, diabetes, lung disease, rheumatoid arthritis, multiple sclerosis, cancer, vitiligo, depression, Parkinson’s disease, heart disease, peripheral arterial disease, osteoporosis, thyroid disease, and stroke

**Fig. 1:**
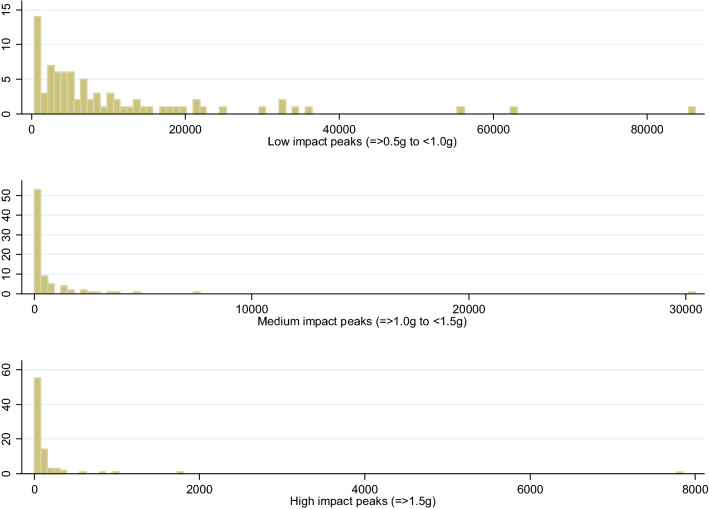
Distribution of physical activity impact counts by levels

Figure 1 shows the distribution of PA counts by the three defined levels. As can be seen, the majority of vertical-impact counts recorded for study participants were within the low-impact PA group. The median number of low-impact PA counts was 5281 (inter-quartile range (IQR) 2516–12,997). The median dropped to 189 (IQR 65–593) for medium-impact PA counts and 39 (IQR 9–105) for high-impact activity counts. A description of the study population lower limb bone and muscle parameters is presented in Table 2.

**Table 2:** Lower limb bone outcomes study population descriptive statistics

	All (*n* = 82)		Men (*n* = 48)		Women (*n* = 34)	
	Mean	SD	Mean	SD	Mean	SD
pQCT parameters						
4% slice						
Total area (mm^2^)	1322.64	204.08	1439.72	162.08	1160.79	133.54
Total density (mg/cm^3^)	274.16	51.12	290.38	46.41	251.75	49.40
Trabecular density (mg/cm^3^)	230.24	45.60	236.98	42.55	220.93	48.62
38% slice						
Cortical density (mg/cm^3^)	1127.35	37.10	1133.14	37.24	1119.34	35.92
Cortical area (mm^2^)	300.82	57.55	336.43	40.70	251.60	37.67
Cortical thickness (mm)	4.84	0.78	5.25	0.65	4.28	0.57
Polar strength strain index (mm^3^)	1842.50	398.91	2084.01	299.57	1508.64	250.51
Total area (mm^2^)	477.74	74.74	521.30	55.98	417.53	52.30

**Fig. 2:**
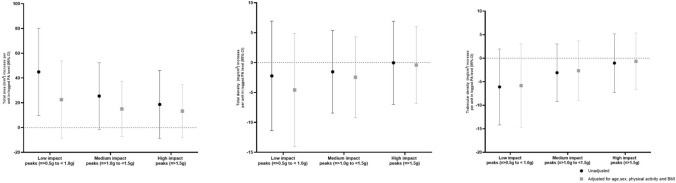
Associations between increase per unit change (95% confidence interval) in physical activity peaks and lower limb bone parameters assessed by pQCT, 4% slice, before and after adjustment for age, sex, BMI, and impact level

At the 4% distal site there were no robust associations between low-, medium-, or high-impact PA counts and volumetric density or bone size (Fig. 2), after adjustment for sex, age, BMI, and PA ((total density 4% slice and low-impact (− 4.58 [− 14.03, 4.88]); medium-impact (− 2.46 [− 9.21, 4.29]); and high-impact (− 0.41 [− 6.85, 6.03])) and (total area 4% slice and low-impact (22.43 [− 8.73, 53.77]); medium-impact (15.08 [− 7.23, 37.40]); and high-impact (13.27 [− 8.02, 34.57])). Results from the linear regression analysis demonstrate that greater low-, medium-, and high-impact PA counts were related to greater total bone area and greater volumetric density with greater high-impact activity counts.

**Fig. 3:**
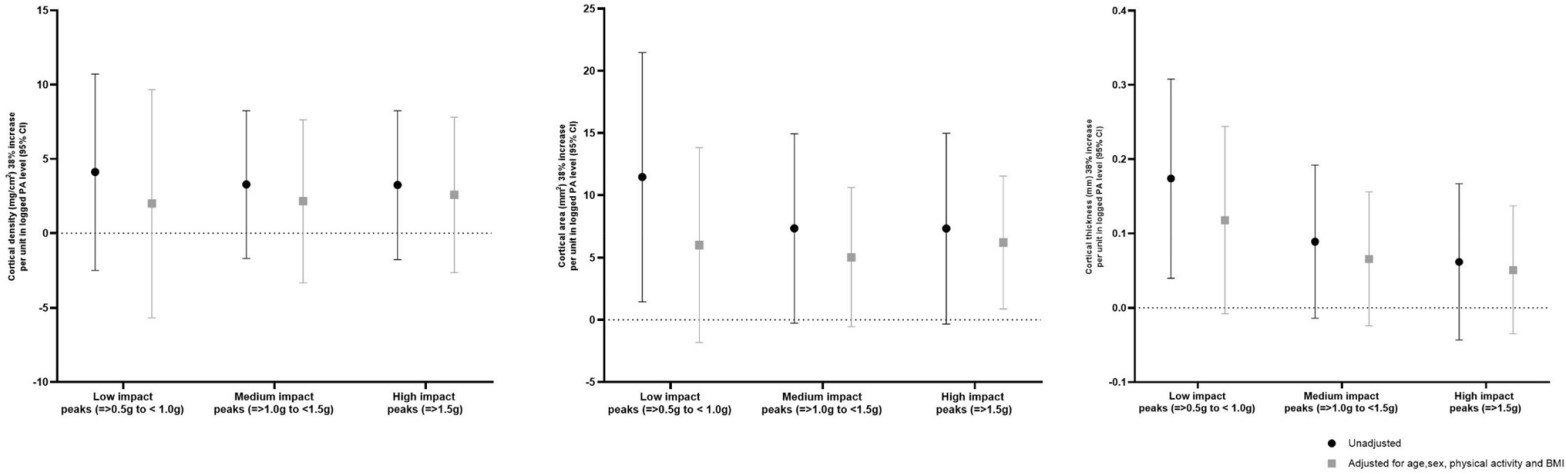
Associations between increase per unit change (95% confidence interval) in physical activity peaks and lower limb bone cortical parameters assessed by pQCT, 38% slice, before and after adjustment for age at pQCT, sex, BMI, and impact level

Positive associations were seen between high-impact PA counts and cortical area at the 38% slice (7.33 [− 0.33, 14.98]), and this association remained after adjustment (6.21 [0.88, 11.54]) (Fig. 3). Positive regression coefficients between low-, medium-, and high-impact PA counts and cortical density (low-impact (4.12 [− 2.48, 10.72]); medium-impact (3.28 [− 1.68, 8.24]); and high-impact (3.24 [− 1.76, 8.24])) and cortical thickness (low-impact (0.74 [0.04, 0.31]); medium-impact (0.09 [− 0.01, 0.19]) (Fig. 3); and high-impact (0.06 [− 0.04, 0.17])) indicating greater high-impact PA counts were related with greater values of cortical density and cortical thickness; however, these results did not reach statistical significance after adjustments for confounders.

**Fig. 4:**
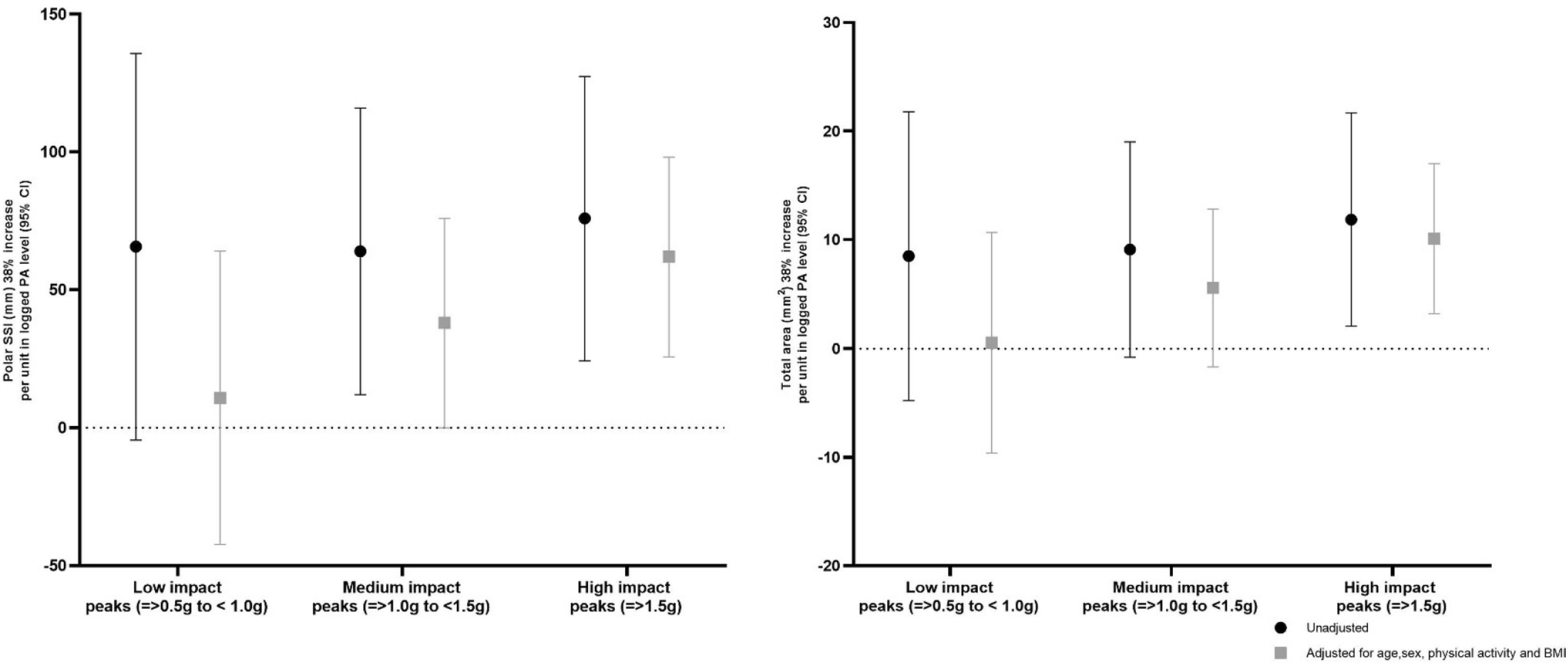
Associations between increase per unit change (95% confidence interval) in physical activity peaks and lower limb bone total area and polar SSI assessed by pQCT, 38% slice, before and after adjustment for age at pQCT, sex, BMI, and impact level

High-impact activity counts were also found to be positively associated with polar SSI (75.85 [24.29, 127.41]) and total area (11.85 [2.03, 21.67]) at the 38% slice and relationships remained after adjustment for age, sex, BMI, and other PA counts ((61.94 [25.73, 98.14]) and (10.09 [3.18, 16.99]), respectively) (Fig. 4).

## Discussion

This study demonstrated most activity peaks recorded in this study population of older adults were at the low-impact (0.5–1.0 g) level, with a median count of only 39 high-impact PA peaks (> 1.5 g) compared to a median count of 5281 low-impact activity peaks. Despite high-activity impact activity being rarely achieved in this study population positive associations were found between high-impact activity counts and cortical area, total area, and polar SSI at the 38% slice. Greater counts of low- and medium-impact PA were also associated with greater cortical density, cortical area, and cortical thickness at the 38% site. However, after adjustments for age, sex, and BMI, no associations remained at the distal 4% (trabecular) site. The site specificity of these observations may be that as individuals’ age, the loss of trabecular bone precedes cortical. The participants in this study were in their eighth decade, by which time we might expect cortical bone loss to be predominating. Extrapolating this to other sites, such as the hip, where fractures occur in cortical sites, it is possible that maintaining PA would positively impact strength here too, at an age where hip fractures predominate.

Several previous studies have described the positive benefits of high-impact PA interventions and improvements in musculoskeletal outcomes [9, 22, 23]. For example, in the LIFTMOR study, following an 8-month, twice weekly, 30-min, supervised high-intensity resistance and impact training (HiRIT) a 2.9% increase in lumbar spine BMD was observed compared to a − 1.2% change in the control group who followed a home-based, low-intensity exercise programme. An increase of 0.3% was also observed at the femoral neck BMD in those in the HiRIT group compared to -1.9% controls [22]. A randomised controlled unilateral intervention study assessing the effects of impact activities, defined as activities that take advantage of body weight impacting the ground, over a 12-month period reported increases in cortical and trabecular bone mineral content at the trochanter and femoral neck [23]. An intervention to promote PA in adults aged 50 to 70 years led to long-term improvements in PA at 12 months; the lasting impact of such behavioural interventions is unclear [24]. Thus, a greater understanding of habitual PA profiles of older adults may help to develop PA guidelines that could be integrated into daily life activities and so may be achievable longer term.

Few studies have monitored habitual levels of PA in older adults and explored the associations with lower limb bone strength. One of these studies is the Cohort for Skeletal Health in Bristol and Avon (COSHIBA) [25]. Using the same accelerometer and processing protocol as applied within this study, most habitual PA counts observed within study participants were defined as low impact (0.5–1.0 g), with participants rarely achieving high-impact counts (> 1.5 g) [25]. Interestingly, in COSHIBA a negative association was observed between low-impact PA and tibia cortical BMD adjusted for age and artefact error grade. Methods are available to translate impact data to daily loading, such as the daily impact stimulus and osteogenic index. Whilst we could not calculate these indices in our study our data agree with previous work in older adults. The PASSWORD study used the osteogenic index [26] and found, as in the current Hertfordshire cohort, that low-impact daily PA may decelerate age-related bone loss and that due to low levels of high-impact activity, this alone could not prevent age-related loss. Together these data that suggest mechanoadaptation of ageing, whereby the set-points of the mechanostat/ sensitivity of osteocytes to changes in strain may adjust to lower levels of loading from muscles. This would mean that the maintenance of even low levels of activity might drive osteocytes response to alter bone remodelling to maintain strength and reduce loss even at lower g strains [27, 28]. Mechanisms of adaptation to reduce bone resorption may be through inhibition of sclerostin and/ or via the RANKL pathway.

In the current study, a threshold of 1.5 g was used to define ‘high’-impact peaks as very few PA peaks were observed in older adults at higher g bands [7]; however, this threshold is greater than the loading achieved by walking, but would be achievable through such activities as an aerobics class designed for older adults [13]. As previously noted, the ‘high’-impact peaks used within this study are much lower than would be seen in younger populations [8], for example, in adolescents a threshold for vertical impacts of 4 g, which are seen during running, was found to be associated with hip BMD [18], and in premenopausal women a positive relationship between hip BMD and vertical-impact activity counts above a 3.9 g [29].

A strength of this study is that physical activity counts were objectively measured using accelerometers, whereas PA data are obtained using questionnaires leading to record inaccurate estimates through recall bias or incorrect estimation of time spent active. Collecting activity data using accelerometers also allows all different impacts of PA to be captured, leading to better understanding habitual daily activities profiles of older adults can help inform future physical activities strategies within this age group.

The main limitation of this study is that a vertical-impact activity count, regardless of impact level, does not directly equate to a step or duration of activity. This means that direct translation of the findings of this, and studies using similar methodologies, into a policy recommendation to prescribe particular exercise levels or direct translation into fracture risk reduction is not possible. Whilst the positive associations between activity counts and bone geometry demonstrated in this study are small, it is likely the effect of greater habitual PA levels might not only be beneficial to bone health but all areas of musculoskeletal health.

A further limitation of this study is the 2-year time gap between collection of PA data and collection of pQCT data. Further longitudinal studies exploring pQCT change and habitual PA profiles in older adults would be required to confirm these findings. Another potential limitation is the healthy survivor bias which is unavoidable in a cohort aged 80 years.

In conclusion, in this relatively healthy cohort of community-dwelling men and women low-impact activity was the predominant level of PA achieved. High-impact PA was associated with greater cortical area and polar SSI. The lack of association at the distal site suggests that the adaptations in older age are at predominately in cortical bone. These results suggest that older adults rarely engage in PA with vertical-impact loads greater than walking; however, even small amounts of high-impact PA may be beneficial for bone health. This study provides insights into habitual mechanical loading of older adults and could be used as evidence to develop strategies to encourage older adults to remain active regardless of the impact level.

## References

[1] Frost HM (2003). Bone's mechanostat: a 2003 update. Anat Rec A Discov Mol Cell Evol Biol.

[2] Frost HM (1987). Bone “mass” and the “mechanostat”: a proposal. Anat Rec.

[3] Van Langendonck L, Lefevre J, Claessens AL, Thomis M, Philippaerts R, Delvaux K, Lysens R, Renson R, Vanreusel B, Vanden Eynde B, Dequeker J, Beunen G (2003). Influence of participation in high-impact sports during adolescence and adulthood on bone mineral density in middle-aged men: a 27-year follow-up study. Am J Epidemiol.

[4] Cleland C, Ferguson S, Ellis G, Hunter RF (2018). Validity of the international physical activity questionnaire (IPAQ) for assessing moderate-to-vigorous physical activity and sedentary behaviour of older adults in the United Kingdom. BMC Med Res Methodol.

[5] Elhakeem A, Hannam K, Deere KC, Hartley A, Clark EM, Moss C, Edwards MH, Dennison E, Gaysin T, Kuh D, Wong A, Fox KR, Cooper C, Cooper R, Tobias JH (2017). Associations of lifetime walking and weight bearing exercise with accelerometer-measured high impact physical activity in later life. Prev Med Rep.

[6] Sember V, Meh K, Sorić M, Starc G, Rocha P, Jurak G (2020). Validity and reliability of international physical activity questionnaires for adults across EU countries: systematic review and meta analysis. Int J Environ Res Public Health.

[7] Deere KC, Hannam K, Coulson J, Ireland A, McPhee JS, Moss C, Edwards MH, Dennison E, Cooper C, Sayers A, Lipperts M, Grimm B, Tobias JH (2016). Quantifying habitual levels of physical activity according to impact in older people: accelerometry protocol for the VIBE study. J Aging Phys Act.

[8] Hannam K, Deere K, Worrall S, Hartley A, Tobias JH (2016). Characterization of vertical accelerations experienced by older people attending an aerobics class designed to produce high impacts. J Aging Phys Act.

[9] Martyn-St James M, Carroll S (2009). A meta-analysis of impact exercise on postmenopausal bone loss: the case for mixed loading exercise programmes. Br J Sports Med.

[10] National Health Service (2018) Exercises for strong bones. https://www.nhs.uk/live-well/exercise/exercises-for-strong-bones/

[11] Morgan GS, Willmott M, Ben-Shlomo Y, Haase AM, Campbell RM (2019). A life fulfilled: positively influencing physical activity in older adults—a systematic review and meta-ethnography. BMC Public Health.

[12] Rai R, Jongenelis MI, Jackson B, Newton RU, Pettigrew S (2020). Factors influencing physical activity participation among older people with low activity levels. Ageing Soc.

[13] Tobias JH, Gould V, Brunton L, Deere K, Rittweger J, Lipperts M, Grimm B (2014). Physical activity and bone: may the force be with you. Front Endocrinol (Lausanne).

[14] Syddall HE, Aihie Sayer A, Dennison EM, Martin HJ, Barker DJ, Cooper C (2005). Cohort profile: the Hertfordshire cohort study. Int J Epidemiol.

[15] Syddall HE, Simmonds SJ, Carter SA, Robinson SM, Dennison EM, Cooper C, Hertfordshire Cohort Study Research G (2019). The Hertfordshire Cohort study: an overview. F1000Res.

[16] Schaap LA, Peeters GM, Dennison EM, Zambon S, Nikolaus T, Sanchez-Martinez M, Musacchio E, van Schoor NM, Deeg DJ (2011). European Project on OSteoArthritis (EPOSA): methodological challenges in harmonization of existing data from five European population-based cohorts on aging. BMC Musculoskelet Disord.

[17] Hannam K, Deere KC, Hartley A, Clark EM, Coulson J, Ireland A, Moss C, Edwards MH, Dennison E, Gaysin T, Cooper R, Wong A, McPhee JS, Cooper C, Kuh D, Tobias JH (2017). A novel accelerometer-based method to describe day-to-day exposure to potentially osteogenic vertical impacts in older adults: findings from a multi-cohort study. Osteoporos Int.

[18] Deere K, Sayers A, Rittweger J, Tobias JH (2012). Habitual levels of high, but not moderate or low, impact activity are positively related to hip BMD and geometry: results from a population-based study of adolescents. J Bone Miner Res.

[19] Elhakeem A, Hannam K, Deere KC, Hartley A, Clark EM, Moss C, Edwards MH, Dennison E, Gaysin T, Kuh D (2018). Physical activity producing low, but not medium or higher, vertical impacts is inversely related to BMI in older adults: findings from a multicohort study. J Gerontol: Ser A.

[20] Laskey MA, de Bono S, Zhu D, Shaw CN, Laskey PJ, Ward KA, Prentice A (2010). Evidence for enhanced characterization of cortical bone using novel pQCT shape software. J Clin densitom.

[21] StataCorp. (2015) Stata Statistical Software: Release 14. StataCorp LP, College Station, TX

[22] Watson SL, Weeks BK, Weis LJ, Harding AT, Horan SA, Beck BR (2018). High-intensity resistance and impact training improves bone mineral density and physical function in postmenopausal women with osteopenia and osteoporosis: the LIFTMOR randomized controlled trial. J Bone Miner Res.

[23] Allison SJ, Poole KES, Treece GM, Gee AH, Tonkin C, Rennie WJ, Folland JP, Summers GD, Brooke-Wavell K (2015). The influence of high-impact exercise on cortical and trabecular bone mineral content and 3D distribution across the proximal femur in older men: a randomized controlled unilateral intervention. J Bone Miner Res.

[24] Hobbs N, Godfrey A, Lara J, Errington L, Meyer TD, Rochester L, White M, Mathers JC, Sniehotta FF (2013). Are behavioral interventions effective in increasing physical activity at 12 to 36 months in adults aged 55 to 70 years? A systematic review and meta-analysis. BMC Med.

[25] Hannam K, Deere KC, Hartley A, Al-Sari UA, Clark EM, Fraser WD, Tobias JH (2017). Habitual levels of higher, but not medium or low, impact physical activity are positively related to lower limb bone strength in older women: findings from a population-based study using accelerometers to classify impact magnitude. Osteoporos Int.

[26] Savikangas T, Sipilä S, Rantalainen T (2021). Associations of physical activity intensities, impact intensities and osteogenic index with proximal femur bone traits among sedentary older adults. Bone.

[27] Ward K (2012). Musculoskeletal phenotype through the life course: the role of nutrition. Proc Nutr Soc.

[28] Compton JT, Lee FY (2014). A review of osteocyte function and the emerging importance of sclerostin. J Bone Jt Surg Am.

[29] Vainionpää A, Korpelainen R, Vihriälä E, Rinta-Paavola A, Leppäluoto J, Jämsä T (2006). Intensity of exercise is associated with bone density change in premenopausal women. Osteoporos Int.

